# A significant change in selective adsorption behaviour for ethanol by flexibility control through the type of central metals in a metal–organic framework†
†Electronic supplementary information (ESI) available. CCDC 1422201 and 1422202. For ESI and crystallographic data in CIF or other electronic format see DOI: 10.1039/c5sc03325j


**DOI:** 10.1039/c5sc03325j

**Published:** 2015-11-05

**Authors:** Masaaki Sadakiyo, Teppei Yamada, Kenichi Kato, Masaki Takata, Hiroshi Kitagawa

**Affiliations:** a Division of Chemistry , Graduate School of Science , Kyoto University , Kitashirakawa-Oiwakecho, Sakyo-ku , Kyoto 606-8502 , Japan . Email: kitagawa@kuchem.kyoto-u.ac.jp; b International Institute for Carbon-Neutral Energy Research (WPI-I2CNER) , Kyushu University , 744 Moto-oka, Nishi-ku , Fukuoka 819-0395 , Japan; c RIKEN SPring-8 Center , 1-1-1 Kouto, Sayo-cho, Sayo-gun , Hyogo 679-5148 , Japan; d Core Research for Evolutional Science and Technology (CREST) , Japan Science and Technology Agency (JST) , 7 Goban-cho, Chiyoda-ku , Tokyo 102-0076 , Japan

## Abstract

Closed–open structural transformations in flexible metal–organic frameworks (MOFs) are of interest for potential applications such as separation, because of their complete selectivity for the adsorption of specific guest molecules. Here, we report the control of the adsorption behaviour in a series of flexible MOFs, (H_2_dab)[M_2_(ox)_3_] (H_2_dab = 1,4-diammoniumbutane, **M** = Fe, Co, Ni, Zn, or Mg), having different central metals with analogous crystal structures. We found that a significant change in the selective adsorption behaviour for EtOH over MeCHO and MeCN is caused by the type of central metals, without changes in the crystal structures of all phases (except the Ni compound). A systematic study of adsorption measurements and structural analyses of the analogous MOFs reveals for the first time that the framework flexibility around the central metals of MOFs is truly related to the selective adsorption behaviour.

## Abstract 2

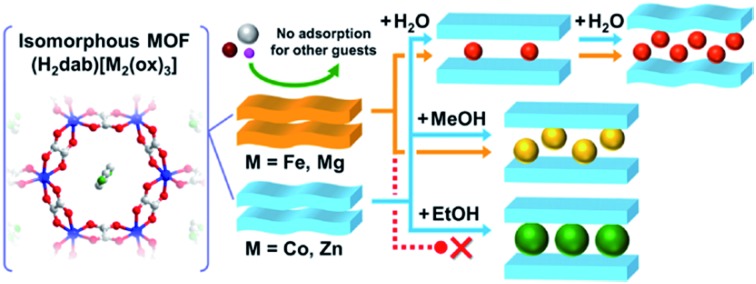
We conducted a systematic study of the control of selective adsorption behaviour through the type of central metals in a series of analogous MOFs that show closed-open structural transformations.

## Introduction

Rational control or intentional modulation of the guest inclusion properties of porous metal–organic frameworks (MOFs) is one of the most important issues for controlling the functionality of these frameworks in applications such as gas storage,^1^,^2^ separation,^3^,^4^ catalysis,^5^ magnetism,^6^,^7^ conductivity,^8^–^11^ and controlled delivery.^12^ Chemically-modifiable MOFs allow changes to be made to the fundamental parameters of the host framework, such as the hydrophilicity,^13^,^14^ acidity or basicity,^15^–^17^ electronic states^18^,^19^ and flexibility,^20^–^23^ in order to afford interactions with target guest molecules. In particular, framework flexibility is a unique feature of MOFs for controlling the adsorption properties. In contrast, other porous materials, for example, porous carbon^24^ or zeolites,^25^ do not show significant framework flexibility during the adsorption/desorption process. Additionally, the flexibility of MOFs often leads to complete selectivity for specific guest molecules, accompanied by a closed–open structural transformation with gate-opening isotherms, which is one of the most effective ways to exclude the adsorption of non-target guest molecules.^22^

We have focused on controlling the selective adsorption behaviour of flexible MOFs that exhibit closed–open structural transformations by means of the difference in the type of central metals in a series of homologous frameworks. We believe that the type of central metals is an important parameter for controlling selective adsorption behaviour because it fine-tunes the energetics of the framework distortion during the adsorption/desorption process. Thus far, some MOFs that show no closed–open behaviour have been investigated for the ability to control adsorption properties through the type of central metals.^26^–^30^ For example, a series of inflexible M_2_(dobdc) (dobdc = 2,5-dioxido-1,4-benzenedicarboxylate, **M** = Mg, Mn, Fe, Co, Ni, and Zn) MOFs has been reported to show different adsorption behaviours for various gases.^26^ However, they did not show significant changes in selective adsorption behaviours because of the rigid framework of the M_2_(dobdc). We believe that a significant change in selective adsorption behaviour could be created in flexible MOFs that show closed–open structural transformations through using different types of central metals, as some research indicates that structural changes in flexible MOFs could be affected by the type of central metals.^31^–^33^ However, significant control of selective adsorption behaviour through the type of central metals, such as adsorption or non-adsorption, has not been observed in homologous frameworks, although an example of TCNQ-based MOFs that contain Zn^2+^ and Mn^2+^ ions and have different structures in the guest-free condition has been reported.^34^ Thus, the effect of the type of central metals on the adsorption properties of isostructural flexible MOFs that show closed–open transformations has not been sufficiently clarified to date.

**Fig. 1:**
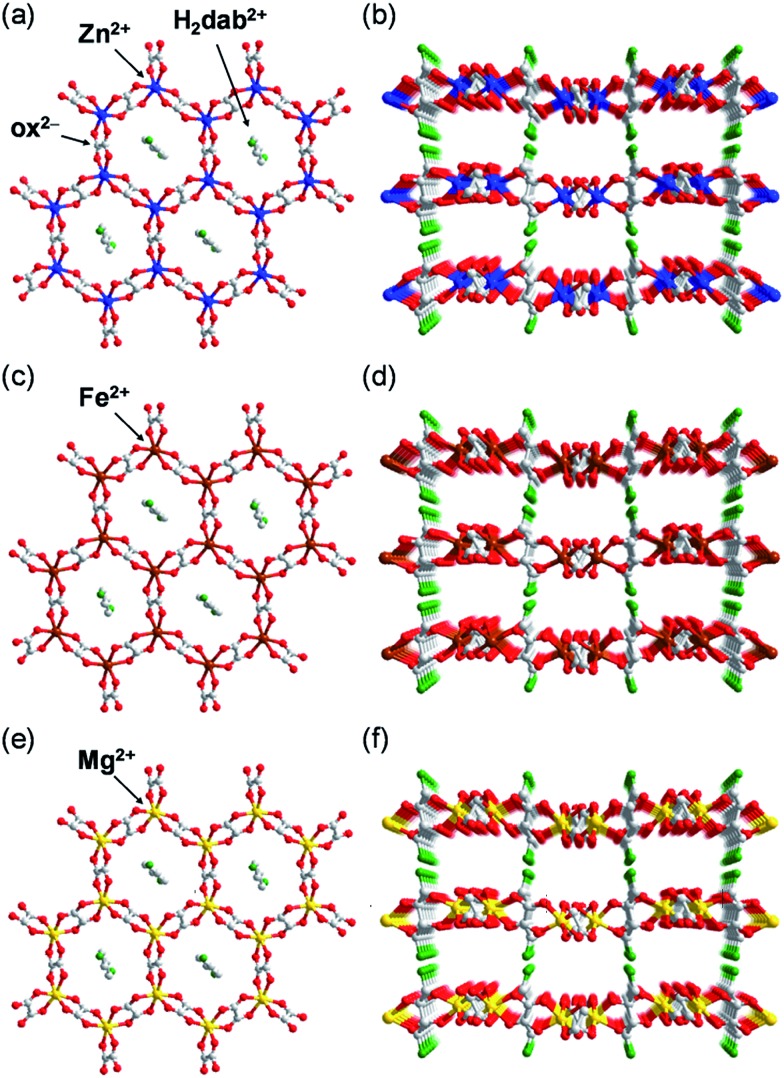
Representation of the crystal structure of **M·6H_2_O**. (a) Honeycomb-shaped layer framework and (b) layered structure of **Zn·6H_2_O**.^35^ (c) Honeycomb-shaped layer framework and (d) layered structure of **Fe·6H_2_O**. (e) Honeycomb-shaped layer framework and (f) layered structure of **Mg·6H_2_O**. Water molecules are omitted. The grey, red, green, blue, brown, and yellow colours correspond to carbon, oxygen, nitrogen, zinc, iron, and magnesium atoms, respectively.

Here, we report a systematic study on the effect of the type of central metals on the selective adsorption behaviour of MOFs that show closed–open structural transformations. We employed an oxalate-bridged layered MOF (H_2_dab)[Zn_2_(ox)_3_]·*n*H_2_O (abbreviated to **Zn·*n*H_2_O**, H_2_dab = 1,4-diammoniumbutane, ox = oxalate), that shows a closed–open structural transformation during its adsorption process (Fig. 1a and b).^35^ This MOF has both hydrogen bond donor (–NH_3_^+^) and acceptor (ox^2–^) sites in the interlayer space; therefore, it can selectively adsorb hydroxyl-functionalized guest molecules such as H_2_O, MeOH, and EtOH over other guests. This MOF was the first material to show complete adsorption selectivity for the large polar guest EtOH over the smaller polar aprotic guests MeCN and MeCHO.^35^ We synthesised a series of MOFs, (H_2_dab)[M_2_(ox)_3_]·*n*H_2_O (**M** = Fe, Co, Ni, Zn, and Mg), having different central metals with almost analogous crystal structures. The guest-free anhydrate states, **M**, also have analogous structures with the exception of the Ni analogue. A systematic study of the adsorption properties of the analogous MOFs reveals that selective adsorption behaviour for EtOH over other guests in the MOF is significantly changed by the type of central metals.

## Experimental section

### Preparation of (H_2_dab)[M_2_(ox)_3_]·*n*H_2_O (**M·*n*H_2_O**)

All chemicals used for the synthesis were purchased as reagent grade. All the samples were hydrothermally synthesised by the reported method.^35^

#### (H_2_dab)[Fe_2_(ox)_3_]·*n*H_2_O (**Fe·*n*H_2_O**)

A mixture of Fe(CH_3_COO)_2_·4H_2_O (10 mmol, 2450 mg), oxalic acid dihydrate (H_2_(ox)·2H_2_O) (40 mmol, 5043 mg), 1,4-diaminobutane (dab) (30 mmol, 3.0 ml), and distilled water (550 mmol, 10 ml) was heated in a 50 ml Teflon-lined bottle. The mixture was heated to 130 °C and was maintained at that temperature for 24 h. It was then slowly cooled to room temperature over 168 h. The reaction temperature was controlled using a programmable oven. The brown coloured crystals were collected by filtration (several crystals were stored in the mother liquid for structural analysis). After washing the samples with distilled water, the samples were dried under air (yield: 1871 mg, 65%). Elemental analysis was performed. (%) calcd for C_10_H_26_N_2_O_18_Fe_2_: C 20.92, H 4.57, N 4.88; found: C 20.94, H 4.51, N 4.88.

#### (H_2_dab)[Co_2_(ox)_3_]·*n*H_2_O (**Co·*n*H_2_O**)

A mixture of Co(CH_3_COO)_2_·4H_2_O (10 mmol, 2491 mg), H_2_(ox)·2H_2_O (20 mmol, 2521 mg), dab (10 mmol, 1.0 ml), and distilled water (550 mmol, 10 ml) was heated in a 50 ml Teflon-lined bottle. The temperature program for the hydrothermal synthesis was the same as that for **Fe·*n*H_2_O**. A rose pink coloured precipitate was collected by filtration. After washing the samples with distilled water, the samples were dried under air (yield: 2661 mg, 92%). Elemental analysis was performed. (%) calcd for C_10_H_18_N_2_O_14_Co_2_: C 23.64, H 3.57, N 5.51; found: C 23.54, H 3.46, N 5.51.

#### (H_2_dab)[Ni_2_(ox)_3_]·*n*H_2_O (**Ni·*n*H_2_O**)

A mixture of Ni(CH_3_COO)_2_·4H_2_O (10 mmol, 2488 mg), H_2_(ox)·2H_2_O (20 mmol, 2521 mg), dab (10 mmol, 1.0 ml), and distilled water (1100 mmol, 20 ml) was heated in a 50 ml Teflon-lined bottle. The temperature program for the hydrothermal synthesis was the same as that for **Fe·*n*H_2_O**. A yellow-green coloured precipitate was collected by filtration. After washing the samples with distilled water, the samples were dried under air (yield: 2462 mg, 97%). Elemental analysis was performed. (%) calcd for C_10_H_18_N_2_O_14_Ni_2_: C 23.66, H 3.57, N 5.52; found: C 23.56, H 3.38, N 5.56.

#### (H_2_dab)[Zn_2_(ox)_3_]·*n*H_2_O (**Zn·*n*H_2_O**)

We previously reported the synthesis of **Zn·*n*H_2_O**.^35^ The protocol for the synthesis was similar to that for **Mg·*n*H_2_O** described below.

#### (H_2_dab)[Mg_2_(ox)_3_]·*n*H_2_O (**Mg·*n*H_2_O**)

A mixture of MgO (10 mmol, 403 mg), H_2_(ox)·2H_2_O (40 mmol, 5043 mg), dab (30 mmol, 3.0 ml), and distilled water (275 mmol, 5 ml) was heated in a 50 ml Teflon-lined bottle. The temperature program for the hydrothermal synthesis was the same as that for **Fe·*n*H_2_O**. Colourless microcrystals were collected by filtration. After washing the samples with distilled water, the samples were dried under air (yield: 2168 mg, 85%). Elemental analysis was performed. (%) calcd for C_10_H_14_N_2_O_12_Mg_2_: C 29.82, H 3.50, N 6.95; found: C 30.08, H 3.53, N 6.98.

### Single-crystal X-ray diffraction

The structures of **Fe·6H_2_O** and **Mg·6H_2_O** were determined by SCXRD for the first time. The structures of **Zn·6H_2_O** and **Zn·2H_2_O** were previously determined and reported.^35^ The data were collected on a Rigaku AFC-7R diffractometer and a Bruker SMART APEXII ULTRA CCD-detector diffractometer using graphite-monochromatic Mo-Kα radiation (*λ* = 0.71073 Å). The SCXRD measurements for **Fe·6H_2_O** and **Mg·6H_2_O** were performed using as-synthesised crystals that were immediately cooled to a low temperature (under N_2_ flow) after being placed on a capillary tube from the mother liquid. The crystal structures were solved using a direct method (SIR2002)^36^ and refined on *F*^2^ using the full-matrix least-squares methods with SHELXL-97.^37^ All of the non-hydrogen atoms were refined using anisotropic thermal factors. In the case of **Fe·6H_2_O**, the hydrogen atoms were refined using isotropic thermal factors.

### X-ray powder diffraction

XRPD measurements were performed using a Bruker D8 ADVANCE (*λ* = 1.54059 Å; Cu-Kα). Synchrotron XRPD measurements were obtained using the BL-8B beamline at the KEK Photon Factory (*λ* = 0.8265 Å) and the RIKEN Materials Science Beamline (BL44B2) at SPring-8 (*λ* = 0.7997 Å).^38^ The samples were sealed under vacuum, H_2_O (approximately 50%, 100% relative pressure), MeOH (100%), and EtOH (100%) conditions after drying at 80 °C under vacuum overnight. The structure of **Zn·4MeOH** was solved by Rietveld refinement and was previously reported.^35^ Pawley or Le Bail fittings were performed using the Materials Studio (Accelrys Inc.) or TOPAS (Bruker AXS Inc.) software package.

### Thermogravimetric analysis

The thermal stability and the adsorbed hydrated phase were evaluated by thermogravimetric analysis (TGA). TG measurements were carried out with Bruker TG-DTA 2000SA under nitrogen gas flow (100 ml min^–1^). The temperature range was from room temperature to 500 °C and the heating rate was 5 °C min^–1^.

### Adsorption measurements

Adsorption/desorption isotherms for N_2_ (77 K), H_2_O, MeOH, MeCN, MeCHO (288 K), EtOH, Me_2_CO, *i*-PrOH, *n*-PrOH, and *n*-BuOH were measured at 298 K using a BELSORP18-PLUS and BELSORP-max (BEL Japan, Inc.). Samples were thoroughly dehydrated by heating at 80 °C overnight.

## Results and discussion

### Syntheses and characterization

Crystals of **Zn·6H_2_O** and **Mg·6H_2_O** were hydrothermally synthesised by heating a mixture of metal oxide (ZnO or MgO), oxalic acid, 1,4-diaminobutane, and distilled water at 130 °C. In the case of **Fe·6H_2_O**, **Co·6H_2_O**, and **Ni·6H_2_O**, metal acetates (M(CH_3_COO)_2_·4H_2_O (**M** = Fe, Co, and Ni)) were used for the reaction instead of metal oxides. Single crystals for X-ray crystallography were successfully obtained for **Zn·6H_2_O**, **Mg·6H_2_O** and **Fe·6H_2_O**. Crystals of **Zn·2H_2_O** were obtained by drying **Zn·6H_2_O** crystals under ambient conditions.

**Fig. 2:**
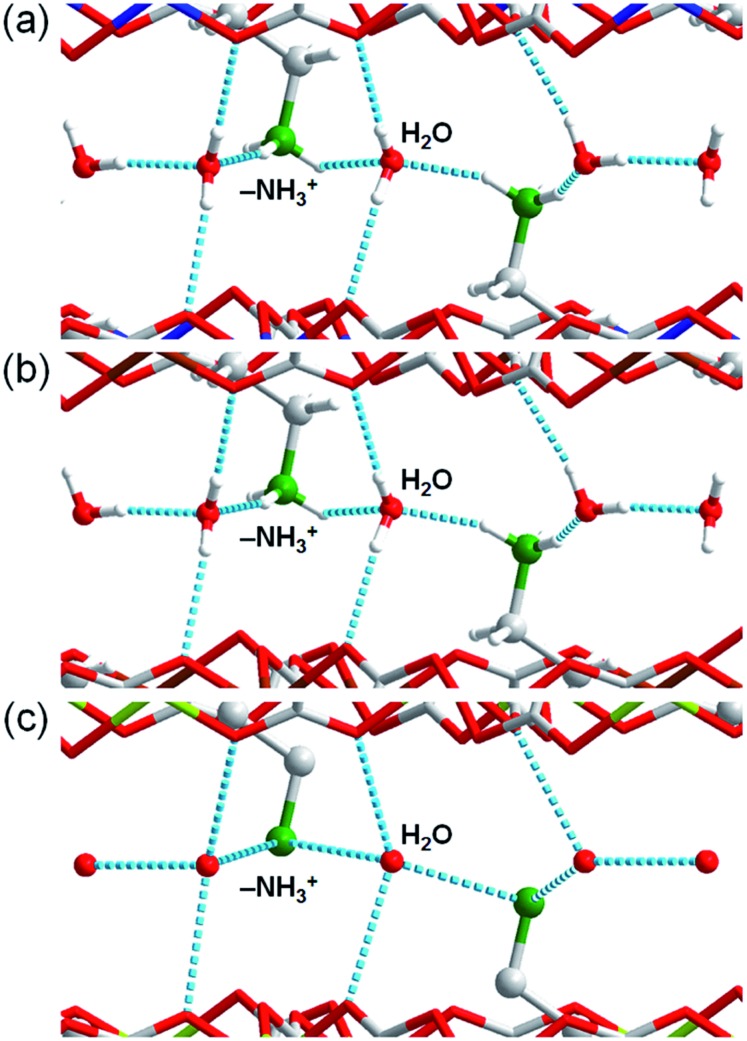
Comparison of the guest arrangements and hydrogen bonds in **M·6H_2_O** (**M** = (a) Zn,^35^ (b) Fe, and (c) Mg). The grey, red, green, blue, brown, and yellow colours correspond to carbon, oxygen, nitrogen, zinc, iron, and magnesium atoms, respectively. The light blue dotted lines denote the hydrogen bonds around the guests.

**Fig. 3:**
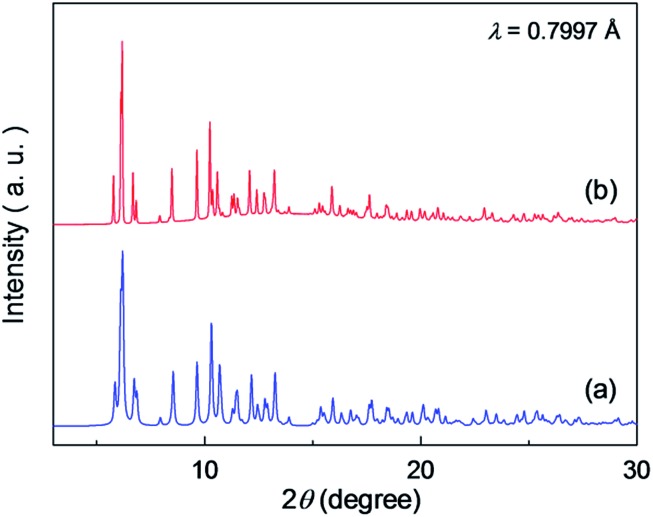
XRPD pattern of the hexahydrate of (a) **Zn·6H_2_O** (simulation)^35^ and (b) **Co·6H_2_O**.

To determine the structures of these MOFs, single-crystal X-ray diffraction (SCXRD) measurements were performed. The crystal structures of **Fe·6H_2_O**, **Zn·6H_2_O** and **Mg·6H_2_O** were successfully determined, and the crystallographic data are shown in Tables 1 and S1–S2.† We previously reported the structure of **Zn·6H_2_O**.^35^ The crystal structures of **Fe·6H_2_O** and **Mg·6H_2_O** were solved using the same space group (*P*2_1_/*n*) as that for **Zn·6H_2_O**. As shown in Fig. 1, there was no apparent difference in the framework structure among the crystals. **Fe·6H_2_O** and **Mg·6H_2_O** also formed the typical honeycomb-shaped layer framework consisting of [M_2_(ox)_3_]^2–^, which incorporated H_2_dab^2+^ ions in the voids as counter cations. The guest water molecules were trapped in the space between the layers. Fig. 2 shows the guest arrangements and the configuration of hydrogen bonds in the interlayer space. The guest water molecules, the oxygen atoms of the ox^2–^ anions, and the ammonium groups of H_2_dab^2+^ are located in the interlayer space and interacted through hydrogen bonds. As is the case with **Zn·6H_2_O**, the guest water molecules were strongly trapped both by the hydrogen bond donor (–NH_3_^+^) and acceptor (O atoms on ox^2–^) sites of the host through three types of hydrogen bonds. One site was between water and the hydrogen bond donor sites of –NH_3_^+^; another site was between water and the hydrogen bond acceptor sites of ox^2–^ ions; and the third site was between neighboring water molecules. Each water molecule formed two hydrogen bonds with both hydrogen bond donor and accepter sites. It should be noted that the guest arrangements and configuration of the hydrogen bonds in **Fe·6H_2_O** and **Mg·6H_2_O** were approximately the same as those in **Zn·6H_2_O**, meaning that the central metals were successfully changed to other elements without significant distortion of the crystal structures. Single crystals of **Co·*n*H_2_O** and **Ni·*n*H_2_O** could not be obtained in this synthesis; however, as shown in Fig. 3, the Co compound shows a similar X-ray powder diffraction (XRPD) pattern to the hexahydrate under humidified conditions, confirming the existence of a hexahydrate **Co·6H_2_O** phase that is isostructural with **Fe·6H_2_O**, **Mg·6H_2_O** and **Zn·6H_2_O**. Note that the **Ni·*n*H_2_O** compound only showed the dihydrate XRPD pattern even under humidified conditions, indicating that there is no **Ni·6H_2_O** phase, as mentioned below.

**Table 1:** Comparison of crystallographic data collection parameters of the SCXRD analysis for **Fe·6H_2_O**, **Zn·6H_2_O**,^35^ and **Mg·6H_2_O**

	**Fe·6H_2_O**		**Zn·6H_2_O**		**Mg·6H_2_O**	
Formula	C_10_H_26_N_2_O_18_Fe_2_		C_10_H_26_N_2_O_18_Zn_2_		C_10_H_26_N_2_O_18_Mg_2_	
Formula weight (g mol^–1^)	574.03		593.07		510.95	
Crystal system	Monoclinic		Monoclinic		Monoclinic	
Space group	*P*2_1_/*n* (no. 14)		*P*2_1_/*n* (no. 14)		*P*2_1_/*n* (no. 14)	
Unit cell dimensions (Å, deg.)	*a* = 8.2586(9)		*a* = 8.3007(8)		*a* = 8.292(2)	
	*b* = 15.864(2)	*β* = 113.190(1)	*b* = 15.660(2)	*β* = 114.592(1)	*b* = 15.688(4)	*β* = 114.800(1)
	*c* = 9.421(1)		*c* = 9.3885(9)		*c* = 9.380(3)	
Volume (Å)	1134.5(2)		1109.7(2)		1107.6(5)	
*Z*	2		2		2	
Calcd density (g cm^–3^)	1.680		1.775		1.532	
Crystal size (mm^3^)	0.30 × 0.25 × 0.08		0.25 × 0.25 × 0.05		0.30 × 0.30 × 0.05	
Temperature (K)	100		100		113	
Wave length (Å)	0.71073 (Mo-Kα)					
Theta range (deg.)	2.57–28.76		2.60–28.64		3.00–27.48	
Reflection collected	6475		12 521		8052	
Unique data/parameters	2699/198		2711/197		2446/146	
*R*_1_/w*R*_2_ (*I* > 2*σ*(*I*))	0.0196/0.0529		0.0169/0.0460		0.0613/0.1980	
*R*_1_/w*R*_2_ (all data)	0.0205/0.0535		0.0175/0.0463		0.0839/0.2502	
GOF	1.048		1.054		1.178	
*μ* (mm^–1^)	1.364		2.248		0.196	

To characterize the hydrated phases and the thermal stabilities of these samples, thermogravimetric analysis (TGA) was performed under nitrogen gas flow. Fig. S1 (ESI†) shows TG curves of air-dried samples of **M·*n*H_2_O** (**M** = Fe, Co, Ni, Zn, and Mg), which showed three-step weight loss at RT, 100–120 °C and 300–350 °C. Considering the chemical compositions of the samples and the temperature regions of the weight losses, the weight losses around RT and 100–120 °C can be attributed to the desorption of included water guests. The mass loss at 100–120 °C corresponded to the desorption of two water molecules per formula unit, indicating the existence of the **M·2H_2_O** dihydrate phase. According to the SCXRD measurement results, it was clear that the transformation from **M·6H_2_O** to **M·2H_2_O** easily occurred at around room temperature and that there are three different hydrated phases consisting of **M·6H_2_O**, **M·2H_2_O** and anhydrate **M**. Note that the Ni compound did not show any weight loss around room temperature, suggesting that it did not have a stoichiometric **M·6H_2_O** phase but only **M·2H_2_O** and **M** phases, which was consistent with the XRPD measurement. The weight losses at approximately 300–350 °C were attributed to the decomposition of ox^2–^ ligands and H_2_dab^2+^, indicating that the framework of (H_2_dab)[M_2_(ox)_3_] can stably exist below 300 °C.

The crystal structure of **Zn·2H_2_O** was successfully determined using SCXRD. As reported in the literature,^35^ the crystal structure of the dihydrate is different from that of the hexahydrate (Fig. S2†). In the dihydrate, the guest water molecules were also bound by the hydrogen bond donor and acceptor sites of the host; however, the **Zn·2H_2_O** had horizontal 2-D layers, whereas the **Zn·6H_2_O** had distorted 2-D layers, indicating that there was a distortion process that occurred during the desorption process.

### Selective adsorption behaviour and structural transformation

To clarify the effect of the type of central metals on the selective adsorption behaviour, adsorption/desorption isotherms were measured using N_2_ (77 K), H_2_O, MeOH, EtOH, MeCN and MeCHO (288 K), Me_2_CO, *i*-PrOH, *n*-PrOH and *n*-BuOH (298 K). The samples were dehydrated by heating at 80 °C under vacuum overnight before the measurements were recorded. The fundamental parameters are shown in Table S3.† 39–44

**Fig. 4:**
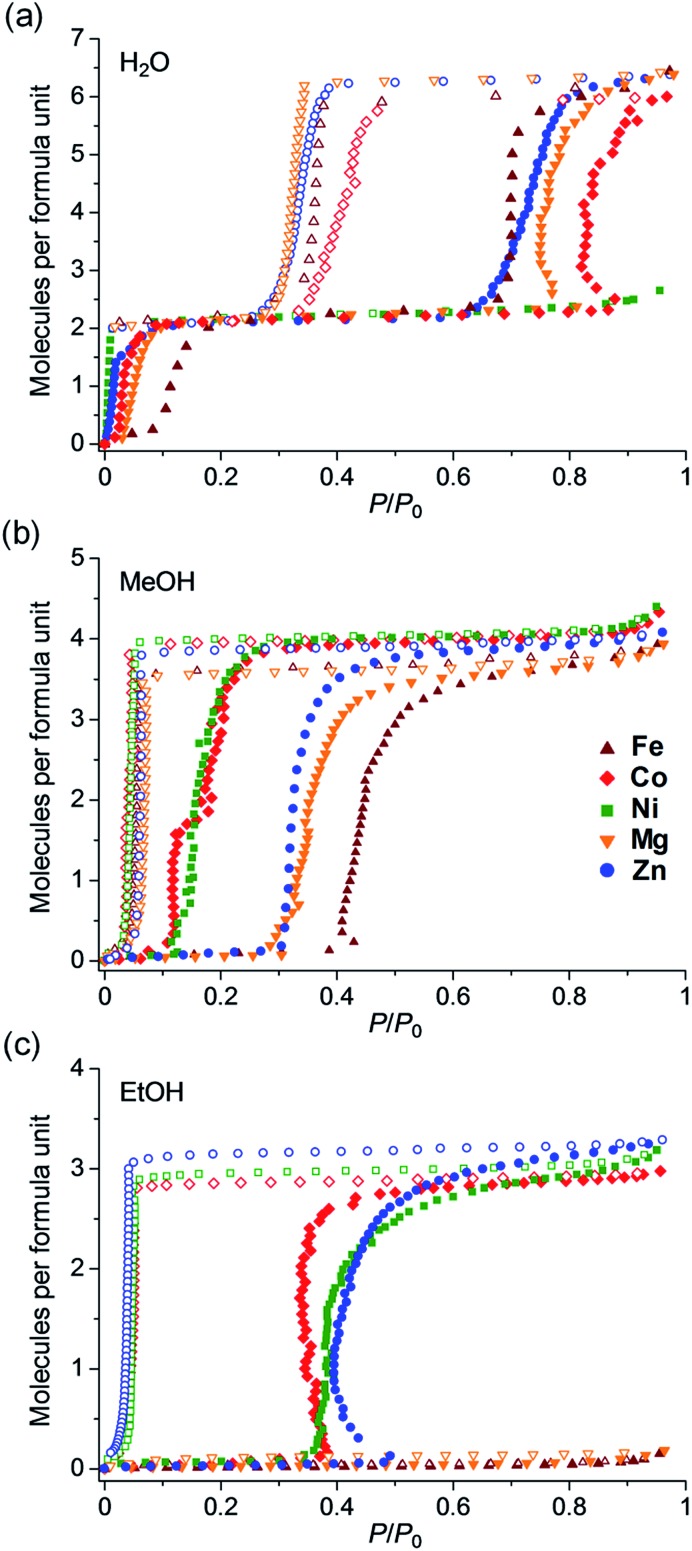
Comparison of adsorption/desorption isotherms of **Fe**, **Co**, **Ni**, **Zn**,^35^ and **Mg** for (a) H_2_O, (b) MeOH and (c) EtOH at 298 K. Brown, red, green, blue, and orange colours correspond to **Fe**, **Co**, **Ni**, **Zn**, and **Mg**, respectively. Filled and open symbols indicate adsorption and desorption isotherms, respectively.

Fig. 4a shows the water vapor adsorption/desorption isotherms, in which all of the samples except the Ni analogue showed two-step hysteric adsorption/desorption isotherms. The first adsorption step below 0.15 *P*/*P*_0_ corresponded to two water molecules, which was attributed to the stoichiometric hydration of **M** to form **M·2H_2_O**. This step confirmed that all of the samples had a dihydrate phase of **M·2H_2_O**, as evidenced by the TGA results. **Fe**, **Co**, **Zn**, and **Mg** showed additional adsorption of four more water molecules at higher humidity (approximately 0.8 *P*/*P*_0_), which was attributed to the transformation from **M·2H_2_O** to **M·6H_2_O**. This result indicated that these samples have three stoichiometric phases: anhydrate, dihydrate, and hexahydrate. Only the Ni did not show any additional adsorption in the high humidity region and only had anhydrate and dihydrate phases. In the case of MeOH adsorption (Fig. 4b), all of the samples showed a large amount of MeOH vapor adsorption with gate-opening isotherms. The amount adsorbed corresponded to four MeOH molecules per formula, indicating a stoichiometric phase of **M·4MeOH**. None of the materials showed a significant change in selective adsorption behaviour, such as non-adsorption of MeOH; however, they showed a clear difference in the gate-opening pressure, which seemed to arise from the type of metal atom. **Fe** and **Mg** showed higher gate-opening pressures than **Zn**, and **Ni** and **Co** showed lower pressures.

In contrast with MeOH adsorption, there was a significant difference in the EtOH adsorption behaviour. Fig. 4c shows the adsorption/desorption isotherms for EtOH vapor. **Co**, **Ni** and **Zn** showed a large amount of EtOH adsorption, which corresponded to three EtOH molecules with typical gate-opening isotherms, whereas **Fe** and **Mg** did not show any apparent EtOH adsorption. This result clearly showed that the difference in the type of central metals caused a significant change in the selective adsorption behaviour for EtOH, resulting in the significant control of EtOH adsorption. As discussed below in the XRPD study, this significant change in the selective adsorption behaviour was purely due to the difference in the framework flexibility as a result of the difference in the type of central metals because these samples have the same crystal structures in all phases (**M**, **M·2H_2_O**, **M·6H_2_O**, **M·4MeOH** and **M·3EtOH**), except in the case of **Ni**. This work is the first systematic study demonstrating the control of selective adsorption behaviour through the type of central metals using flexible MOFs that show closed–open structural transformations. As discussed below, we believe that the difference in adsorption behaviour was derived from the covalent character of the Zn^2+^ and Co^2+^ ions which make the framework more flexible during the adsorption process.

**Fig. 5:**
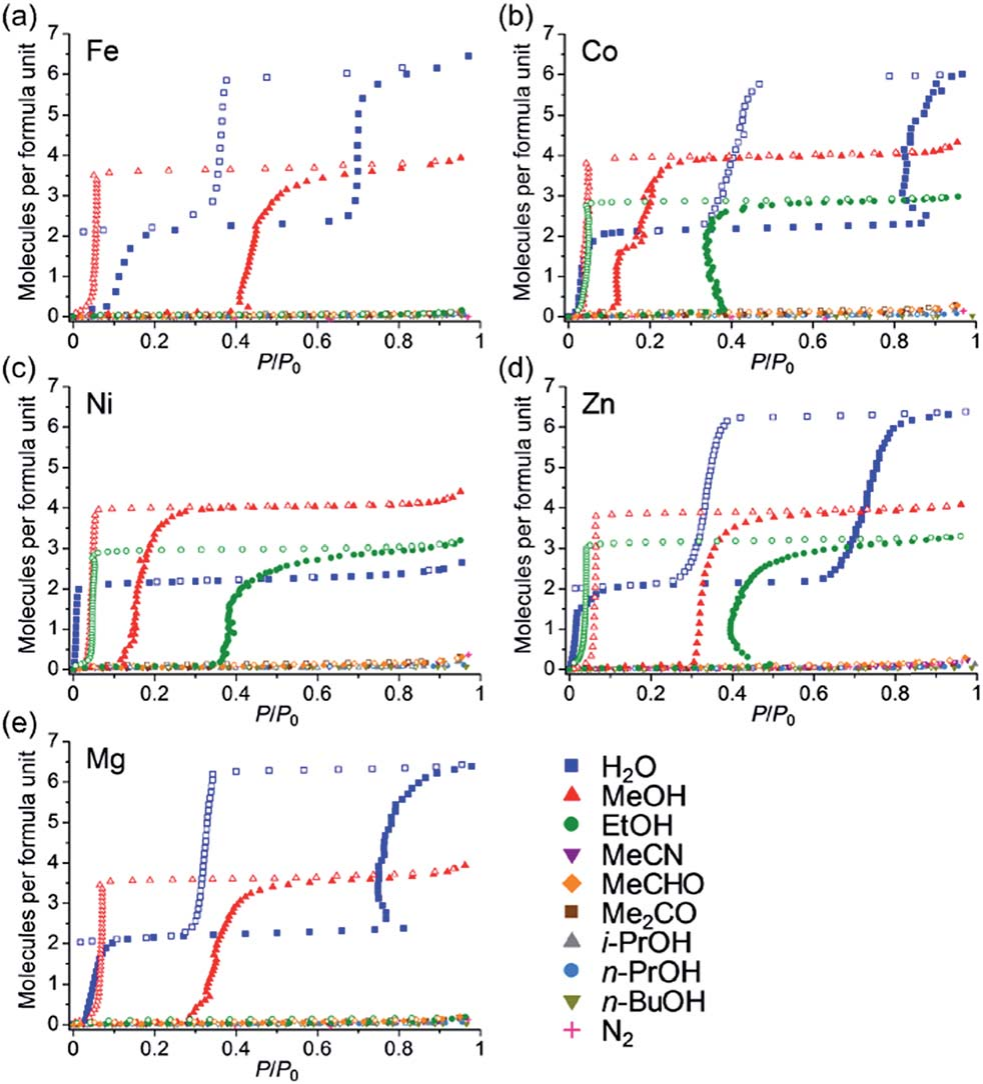
Adsorption/desorption isotherms of (a) **Fe**, (b) **Co**, (c) **Ni**, (d) **Zn**,^35^ and (e) **Mg** for H_2_O, MeOH, EtOH, MeCN and MeCHO (288 K), Me_2_CO, *i*-PrOH, *n*-PrOH, *n*-BuOH and N_2_ (77 K) at 298 K.

Fig. 5 shows the adsorption isotherms for all of the guests. All of the samples did not show significant adsorption of N_2_, MeCN, MeCHO, Me_2_CO, *i*-PrOH, *n*-PrOH and *n*-BuOH. Almost no adsorption of N_2_ (77 K) indicated that the anhydrate phases did not have any apparent microporosity, which confirmed that the adsorption processes for H_2_O, MeOH and EtOH are attributable to closed–open adsorption behaviour. As we previously reported, **Zn** has excellent hydroxyl group recognition properties, particularly, a non-size selective adsorption for polar protic guests (EtOH over MeCN and MeCHO).^35^ Considering that **Co**, **Ni**, and **Zn** did not show any adsorption for aprotic guests such as MeCN and MeCHO despite the fact that these molecules are smaller than EtOH, the Co and Ni also displayed hydroxyl group recognition. All of the samples did not adsorb guest molecules larger than EtOH (*i*-PrOH, *n*-PrOH, and *n*-BuOH), indicating that a size limit of included guest molecules exists.

To clarify the structural transformation during the adsorption processes of these samples, XRPD measurements were performed under various environmental conditions: vacuum (for **M**), exposure to water (approx. 0.5 and 1 *P*/*P*_0_ for **M·2H_2_O** and **M·6H_2_O**), methanol (approx. 1 *P*/*P*_0_ for **M·4MeOH**) and ethanol (approx. 1 *P*/*P*_0_ for **M·3EtOH**). The samples were placed inside a sealed glass capillary, dehydrated by heating at 80 °C overnight and then exposed to the desired guests. The XRPD patterns under these conditions and the cell parameter refinement results by fitting using the Pawley or Le Bail methods are shown in Fig. S3–S11 and Tables S4–S7 (ESI†). As shown in Fig. S3,† the anhydrate phases of **Fe**, **Co**, **Mg**, and **Zn** showed similar patterns, which were all successfully fitted to the same unit cell with a *P*2_1_/*c* space group (Fig. S4, Table S4†), confirming that they had the same structure. **Fe** showed some additional peaks below 5° that could not be fitted by this unit cell. We believe that the diffraction peaks were derived from some superlattice structure of the Fe compound, but not from impurities because these peaks also show changes due to exposure to guests (Fig. S5, S7, and S9†). Only the **Ni** showed a different pattern, indicating that it had a different crystal structure in the anhydrate phase. In the case of **M·2H_2_O** (Fig. S5†), all of the compounds showed XRPD patterns similar to the **Zn·2H_2_O**, indicating that the difference in the type of central metals did not cause significant structural changes in the dihydrate state, as was the case with the hexahydrate phases discussed above. These dihydrate patterns were successfully fitted to the same unit cell with a space group of *P*1 (Fig. S6, Table S5†), which was different from that of **M**, confirming that the adsorption process from **M** to **M·2H_2_O** included a structural transformation. Note that an additional peak existed below 5° in **Fe·2H_2_O**, which was likely derived from the superlattice structure. As evidenced in the SCXRD results, the hexahydrate phases of the samples showed XRPD patterns similar to **Zn·6H_2_O** (Fig. S7†). The superlattice peaks of the Fe compound disappeared in the hexahydrate phase. These patterns were well fitted using the space group *P*2_1_/*n* (Fig. S8, Table S6†), which was different from the anhydrate and dihydrate, showing that the water adsorption process included two different structural transformations. It should also be noted that there is no prior report of a series of MOFs having different central metals with such structural similarity for each phase during a gate-opening adsorption process, although an example of analogous TCNQ-based MOFs that contain Zn^2+^ and Mn^2+^ ions and have amorphous structures in the guest-free condition has been reported.^34^

**Fig. 6:**
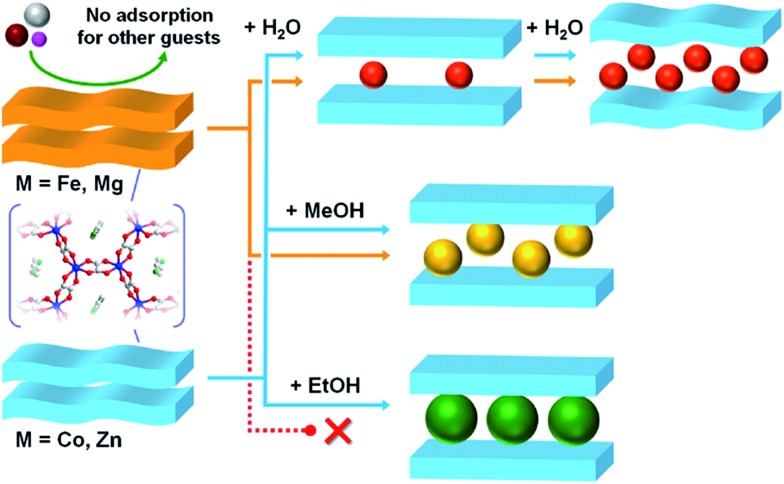
A schematic illustration of the difference in the adsorption behaviour among the homologous **Fe**, **Co**, **Zn**, and **Mg**.

Fig. S9† shows the XRPD patterns of **M·4MeOH**. We previously succeeded in determining the crystal structure of **Zn·4MeOH** (Fig. S12†).^35^ The XRPD patterns were fitted using the same unit cell having a *P*1 space group (Fig. S10, Table S7†). This result showed that the same structural transformation process existed from **M** to **M·4MeOH** during MeOH adsorption for all of the analogues, with the exception of the Ni compound. This result was consistent with the adsorption measurement results, which showed typical closed–open hysteric adsorption isotherms. Considering that the **Fe**, **Mg**, **Co**, and **Zn** compounds had isostructural **M** and **M·4MeOH** phases, the difference in the gate-opening pressure was derived from the difference in the framework flexibility during the adsorption process. This result implied that the host frameworks of **Co** and **Zn** were more flexible than **Fe** and **Mg** during the adsorption process. In the case of the Ni compound, the framework flexibility cannot be discussed in the same manner as the other compounds because it had a different crystal structure in its anhydrate phase of **Ni**. However, to compare the gate-opening pressure, we can hypothesize that the summation of the energy loss due to the structural transformation and the energy gain due to the hydrogen bond formation in Ni compound were similar to those in the Co compound. In the case of **M·3EtOH**, the Co, Ni, and Zn compounds showed almost the same XRPD patterns, indicating that they were also isostructural after EtOH adsorption (Fig. S11†). The patterns were similar to those of **M·4MeOH** and different from **M**. Clearly, the adsorption process from **M** to **M·3EtOH** included an apparent structural transformation, which was similar to the transformation of **M** to **M·4MeOH**. From these results, we could summarize the structural transformations and the difference in selective adsorption behaviours among the homologous MOFs as in Fig. 6. Considering the fact that **Fe** and **Mg**, which were estimated to have lower flexibilities in this transformation, did not show any apparent adsorption of EtOH, we can conclude that the significant change in the selective adsorption behaviour for EtOH through the type of central metals was caused by the change in the framework flexibility. To the best of our knowledge, this is the first report proving that the selective adsorption behaviour of MOFs that show closed–open structural transformations can be controlled by controlling their flexibility through the type of central metals. We next investigated which parameters of the metal ions contributed to the control of the adsorption behaviour. Fe^2+^ and Mg^2+^ ions (in case of no EtOH adsorption) could not be distinguished from Zn^2+^ and Co^2+^ ions (EtOH adsorption) by the order of the fundamental parameters, such as ionic radius (Fe^2+^ (0.78 Å for octahedral coordination) > Co^2+^ (0.75 Å) > Zn^2+^ (0.74 Å) > Mg^2+^ (0.72 Å)), average bond length of M–O (Fe^2+^ (2.121 Å in **M·6H_2_O**) > Zn^2+^ (2.087 Å) > Mg^2+^ (2.075 Å)), and cell volume (Fe (448.9 Å^3^ per formula (see Table S4†)) > Co (444.7 Å^3^) > Mg (443.2 Å^3^) > Zn (442.5 Å^3^)). However, these ions can be distinguished by the order of the complex formation constants (*β*_1_ and *β*_2_) for the ox^2–^ ligand (Co^2+^ (log *β*_1_ = 3.33, log *β*_2_ = 6.20) ≥ Zn^2+^ (log *β*_1_ = 3.42, log *β*_2_ = 6.16) > Fe^2+^ (log *β*_1_ = 3.05, log *β*_2_ = 5.15) > Mg^2+^ (log *β*_1_ = 2.18, no data for log *β*_2_)),^45^ implying that the significant change in adsorption behaviour was related to the chemical bond between the central metal ions and the ox^2–^ ligands. According to the value of ionic potentials (≡ ion charge divided by ionic radius) of these samples (Mg^2+^ (2.78) > Zn^2+^ (2.70) > Co^2+^ (2.67) > Fe^2+^ (2.56)), Mg^2+^ has a strongest electrostatic interaction to oxalate ions. However, Mg^2+^ has a lower complex formation constant for the ox^2–^ ligands than Zn^2+^ and Co^2+^, indicating that there is a high contribution of covalent character of Zn^2+^ and Co^2+^ to the chemical bond with ox^2–^ ligands. We believe that the covalent character of the Co^2+^ and Zn^2+^ ions tended to allow a slight deformation of the surrounding ox^2–^ ions during the gate-open adsorption process, making **Zn** and **Co** more flexible than **Mg** and **Fe**. We also believe that the significant control of selective adsorption behaviour through the type of central metals in this compound was realised because of the existence of framework distortion in the honeycomb layer of [M_2_(ox)_3_]^2–^, as was the case for our compound. [M_2_(ox)_3_]^*n*–^ sometimes forms undulating layered structures accompanied by framework distortion (*e.g.*, **M·6H_2_O**)^46^ but normally shows a flattened framework (*e.g.*, **M·2H_2_O**).^47^–^49^ The difference in the type of central metals seemed to cause differences in the ease of such framework distortion. This type of slight change in the framework structure during the adsorption process might be necessary for achieving the significant change in selective adsorption behaviour.

## Conclusions

In conclusion, we demonstrated the control of selective adsorption behaviour through the type of central metals. We successfully synthesised isostructural frameworks of (H_2_dab)[M_2_(ox)_3_] that showed selective adsorption for hydroxyl-functionalized guests (H_2_O, MeOH, and EtOH). Difference in the type of central metals significantly affects adsorption behaviour for EtOH because of the induced differences in the framework flexibility. There was a tendency for **Fe** and **Mg** to be less flexible than **Zn** and **Co**. We conducted a systematic study of the control of selective adsorption behaviour through the type of central metals in a series of analogous MOFs that show closed–open structural transformations. This study is an important example of the selective adsorption property of MOFs and provides a new opportunity to achieve significant control of selective adsorption behaviour using flexible MOFs.

## Supplementary Material

Supplementary information

Crystal structure data
