# Supplementary material for: A significant change in selective adsorption behaviour for ethanol by flexibility control through the type of central metals in a metal–organic framework
Source: Chem Sci. 2015 Nov 5;7(2):1349–56. doi: 10.1039/c5sc03325j (PMC5975919; doi:10.1039/c5sc03325j)
Supplement: Supplementary file 1 [file SC-007-C5SC03325J-s001.pdf]

Electronic Supplementary Material (ESI) for Chemical Science.
This journal is © The Royal Society of Chemistry 2015

Electronic Supplementary Information

A Significant Change in Selective Adsorption Behaviour for Ethanol by Flexibility Control through the Type of Central Metals in a Metal–Organic Framework

Masaaki Sadakiyo,*[†,‡] Teppei Yamada,[†,||] Kenichi Kato,[§] Masaki Takata,[§] Hiroshi Kitagawa*[†,#]

[†] *Division of Chemistry, Graduate School of Science, Kyoto University, Kitashirakawa-Oiwakecho, Sakyo-ku, Kyoto 606-8502, Japan.*

[‡] *International Institute for Carbon-Neutral Energy Research (WPI-I2CNER), Kyushu University, 744 Moto-oka, Nishi-ku Fukuoka 819-0395, Japan.*

[§] *RIKEN SPring-8 Center, 1-1-1 Kouto, Sayo-cho, Sayo-gun, Hyogo 679-5148, Japan.*

[#] *Core Research for Evolutional Science and Technology (CREST), Japan Science and Technology Agency (JST), 7 Goban-cho, Chiyoda-ku, Tokyo 102-0076, Japan.*

Present address: [||] *Center for Molecular Systems (CMS), Department of Chemistry and Biochemistry, Graduate School of Engineering, Kyushu University, Moto-oka 744, Nishi-ku, Fukuoka, 819-0395, Japan.*

E-mail: kitagawa@kuchem.kyoto-u.ac.jp

Table S1 Atomic coordinates (x 10^4) and equivalent isotropic displacement parameters (Å^2 x 10^3) for **Fe·6H$_2$O**.

	x	y	z	U(eq)
Fe(1)	5275(1)	6700(1)	9580(1)	12(1)
O(4)	2476(1)	6774(1)	8360(1)	16(1)
O(3)	5454(1)	7668(1)	11214(1)	15(1)
O(2)	5043(1)	7490(1)	7729(1)	14(1)
O(1)	8040(1)	6885(1)	10656(1)	15(1)
O(6)	5124(1)	5842(1)	11231(1)	16(1)
O(8)	7462(1)	6773(1)	6639(1)	19(1)
O(5)	5349(1)	5529(1)	8518(1)	16(1)
O(9)	614(1)	5984(1)	9977(1)	19(1)
O(7)	4608(2)	6210(1)	13973(1)	43(1)
N(1)	6937(1)	9976(1)	7719(1)	16(1)
C(5)	5783(2)	10099(1)	9789(1)	17(1)
C(2)	2002(1)	7208(1)	7159(1)	12(1)
C(3)	4937(1)	5088(1)	10790(1)	13(1)
C(4)	5452(2)	9782(1)	8186(1)	18(1)
C(1)	3495(1)	7642(1)	6811(1)	12(1)

Table S2 Atomic coordinates (x 10^4) and equivalent isotropic displacement parameters ($Å^2$ x 10^3) for **Mg·6H_2O**.

	x	y	z	U(eq)
Mg(1)	-291(1)	1693(1)	341(1)	10(1)
O(7)	-10398(5)	-1216(2)	-6013(4)	43(1)
O(1)	1984(3)	3136(1)	4261(3)	12(1)
O(6)	100(3)	-834(1)	1259(3)	13(1)
O(5)	-368(3)	562(1)	1446(3)	13(1)
O(9)	-4388(3)	-1001(2)	23(3)	17(1)
O(2)	-73(3)	2485(1)	2168(3)	12(1)
O(8)	-7544(3)	-1836(2)	-3327(3)	18(1)
O(3)	4538(3)	2363(1)	3710(3)	13(1)
O(4)	2460(3)	1788(1)	1518(3)	13(1)
N(1)	-6960(4)	-9(2)	-2759(3)	15(1)
C(1)	1481(4)	2653(2)	3094(4)	10(1)
C(2)	2971(4)	2227(2)	2743(4)	10(1)
C(3)	-77(4)	-78(2)	787(4)	11(1)
C(4)	-5452(5)	199(2)	-3179(4)	17(1)
C(5)	-5793(5)	-110(2)	-4819(4)	16(1)

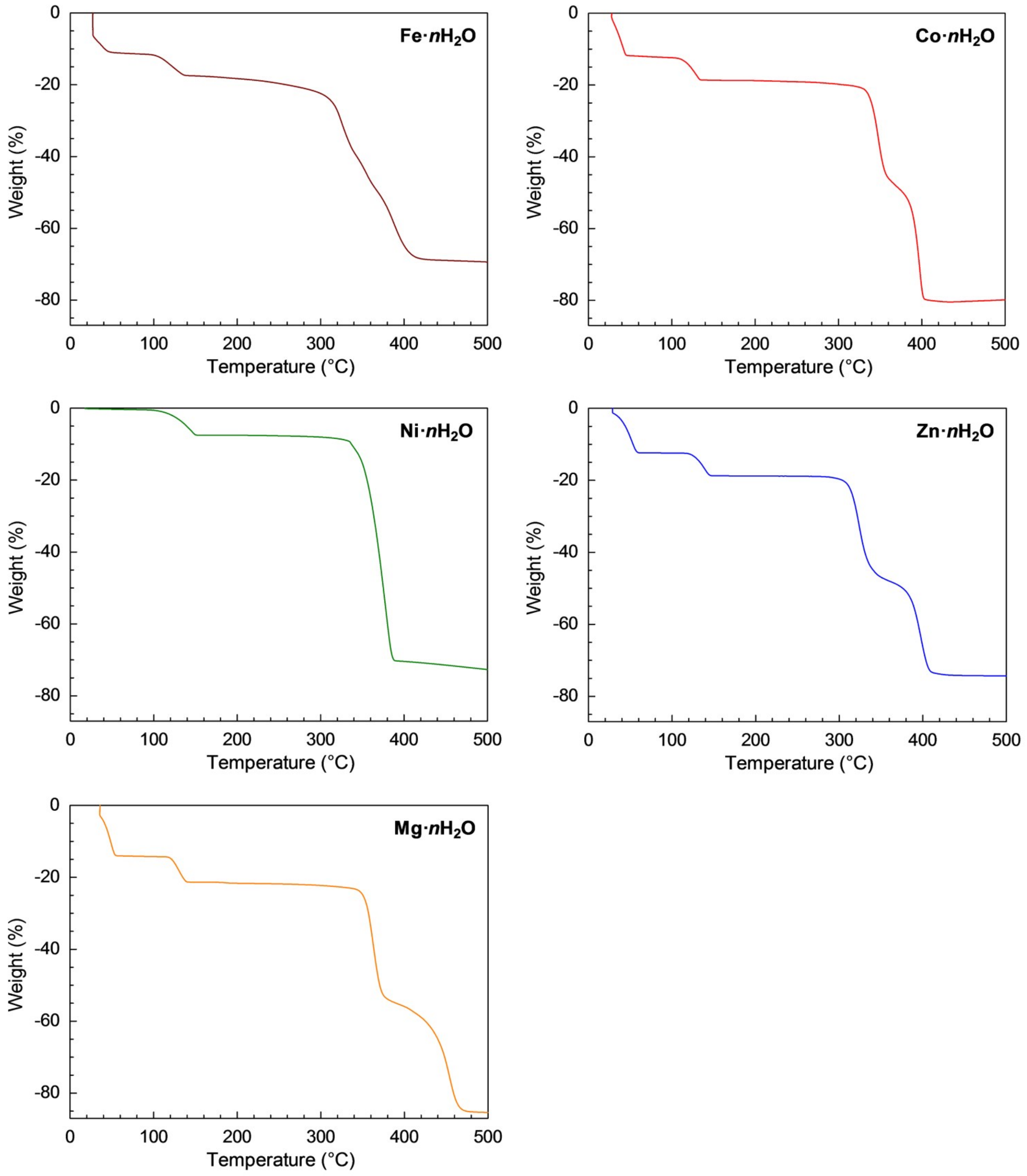

Figure S1 TGA curves of air-dried **M·nH$_2$O** (M = Fe, Co, Ni, Zn,[1] Mg) from RT to 500 °C under N_2 gas flow condition.

Table S3 Fundamental parameters of the adsorbents. "P" and "A" in proticity indicate "protic" and "aprotic", respectively.

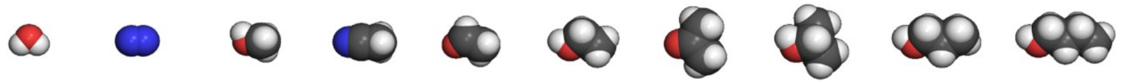

	H_2O	N_2	MeOH	MeCN	MeCHO	EtOH	Me_2CO	*i*−PrOH	*n*−PrOH	*n*−BrOH
Kinetic diameter (Å)	2.64−2.9	3.64−3.80	3.626−4.0	4.3	4.5	4.3−4.53	4.6−4.7	4.7	4.7	5.0
Dipole moment (D)	1.86	0	1.69	3.81	2.64	1.71	2.82	1.56	1.49	1.52
Proticity	P	A	P	A	A	P	A	P	P	P

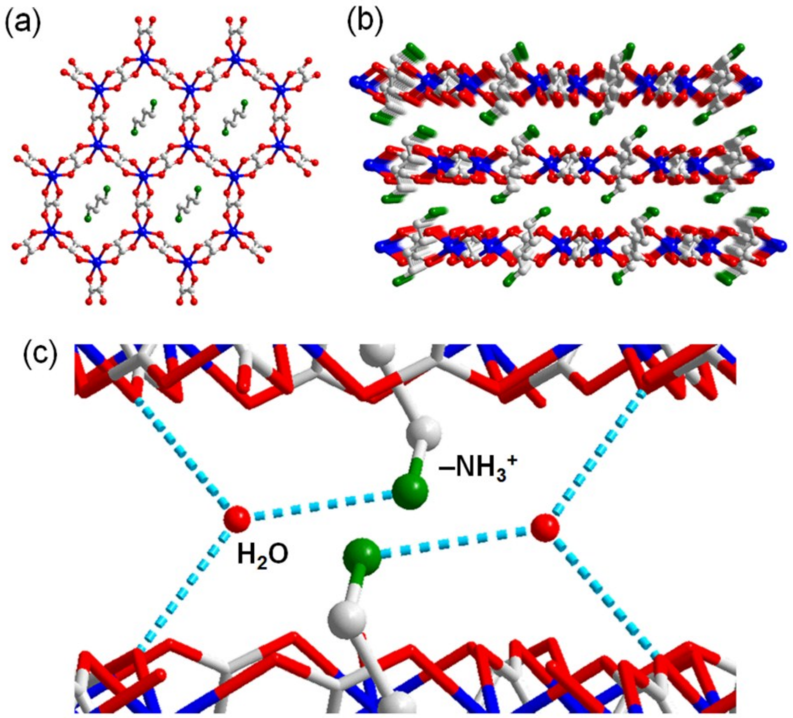

Figure S2 Crystal structure of **Zn·2H$_2$O**.[1] (a) Honeycomb layer framework of $[Zn_2(ox)_3]^{2-}$ (b) A perspective view along a layer. The guest molecules have been omitted. (c) Hydrogen bonds around the guest molecules (Light blue dotted lines).

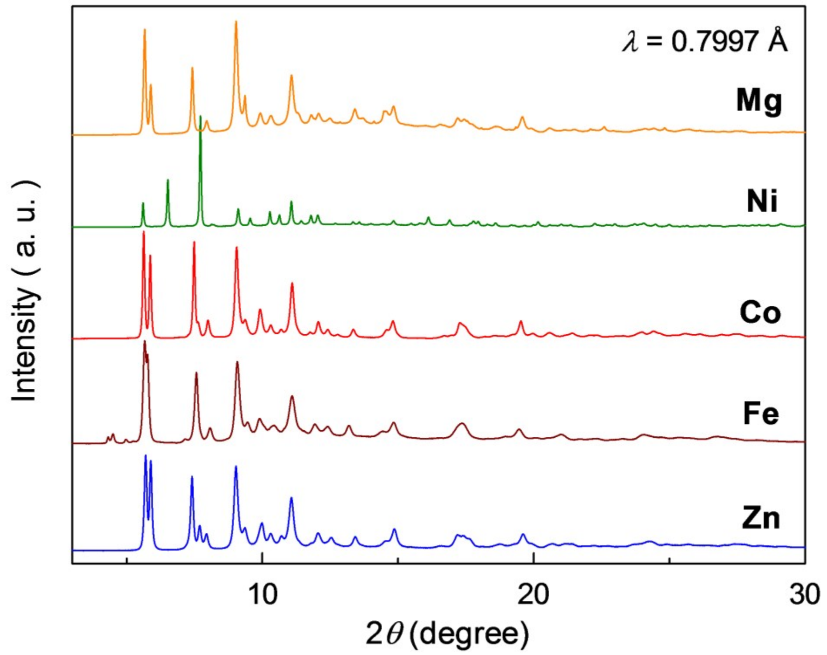

Figure S3 XRPD patterns of anhydrate of **M** (M = Fe, Co, Ni, Zn, and Mg) at RT.

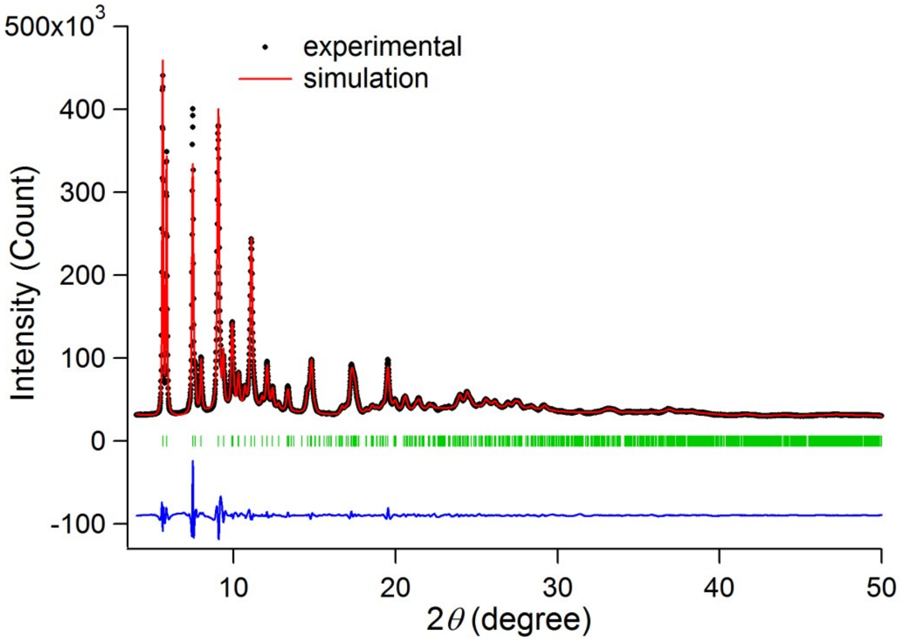

Figure S4 Results of Le Bail fittings for anhydrate exemplified by **Co**.

Table S4. Refined cell parameters of anhydrate of **M** (M = Fe, Co, Zn,[1] and Mg) at RT.

	a / Å	b / Å	c / Å	α / °	β / °	γ / °	Space group	R_{wp}	Volume	Volume per formula
Fe	6.0910(5)	16.195(2)	9.152(1)	90	96.103(5)	90	$P2_1/c$	2.51%	897.8	448.9
Co	6.1564(3)	16.260(1)	8.9399(6)	90	96.373(3)	90	$P2_1/c$	3.67%	889.4	444.7
Zn	6.2150(3)	16.0414(8)	8.9277(5)	90	96.122(1)	90	$P2_1/c$	2.23%	885.99	442.5
Mg	6.2014(4)	16.144(1)	8.9049(7)	90	96.201(4)	90	$P2_1/c$	2.98%	886.3	443.2

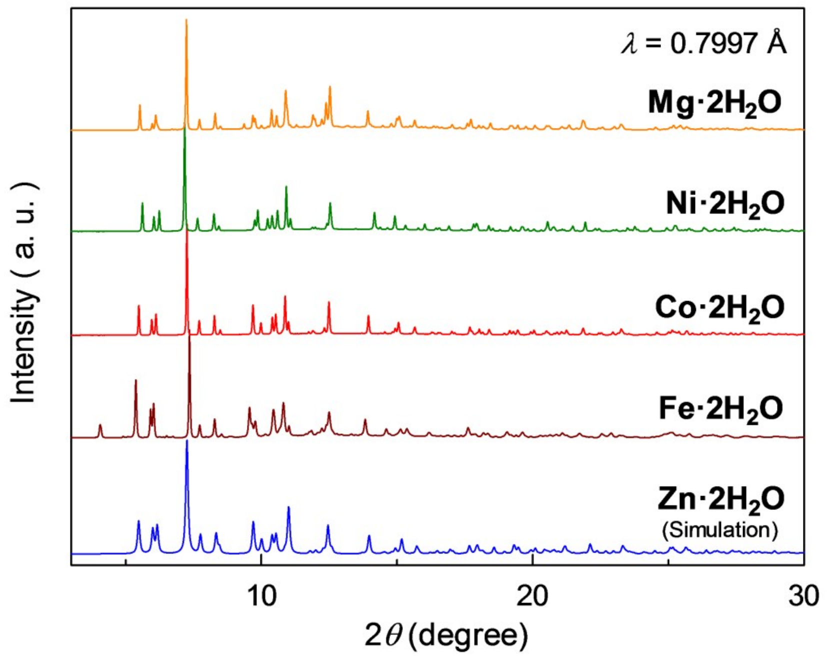

Figure S5 XRPD patterns of dihydrate of **M·2H$_2$O** (M = Fe, Co, Ni, Zn (simulation),[1] and Mg) at RT.

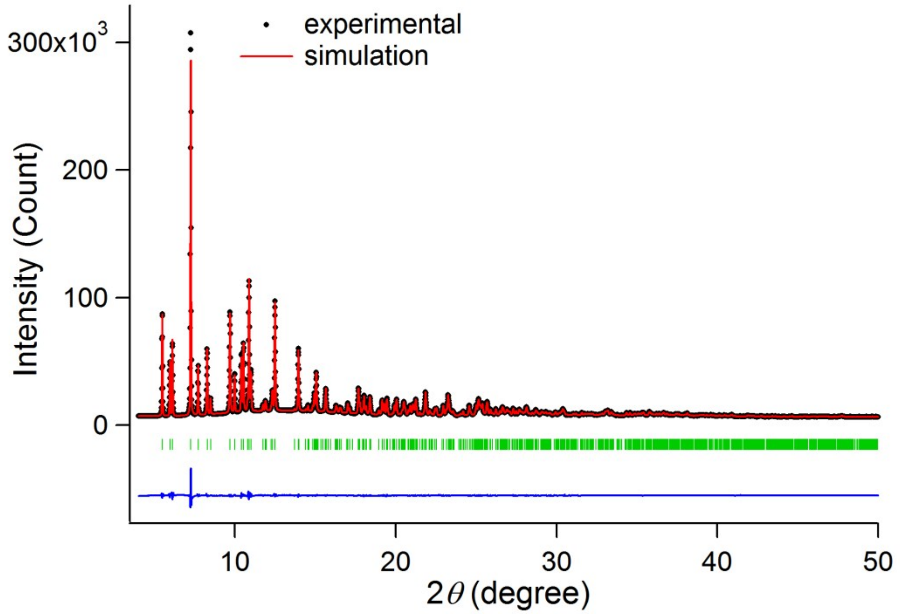

Figure S6 Results of Le Bail fittings for dihydrate exemplified by **Co·2H$_2$O**.

Table S5 Refined cell parameters of anhydrate of **M·2H$_2$O** (M = Fe, Co, Ni, Zn,[1] and Mg) at RT.

	a / Å	b / Å	c / Å	α / °	β / °	γ / °	Space group	R_{wp}	Volume	Volume per formula
Fe·2H$_2$O	6.6534(3)	9.0908(4)	9.6519(5)	63.040(3)	87.747(3)	70.963(3)	P-1	4.69%	487.79	487.79
Co·2H$_2$O	6.7316(1)	8.9944(1)	9.4991(1)	62.503(1)	88.026(1)	71.209(1)	P-1	2.33%	478.48	478.48
Ni·2H$_2$O	6.8394(1)	8.8899(1)	9.3270(1)	62.146(1)	88.322(1)	70.808(1)	P-1	1.87%	468.26	468.26
Zn·2H$_2$O	6.7613(6)	8.9907(8)	9.4623(9)	62.799(1)	87.997(1)	71.109(2)	P-1	2.31%	479.65	479.65
Mg·2H$_2$O	6.7368(2)	8.9925(3)	9.4303(3)	62.575(2)	88.124(2)	71.486(2)	P-1	5.00%	476.45	476.45

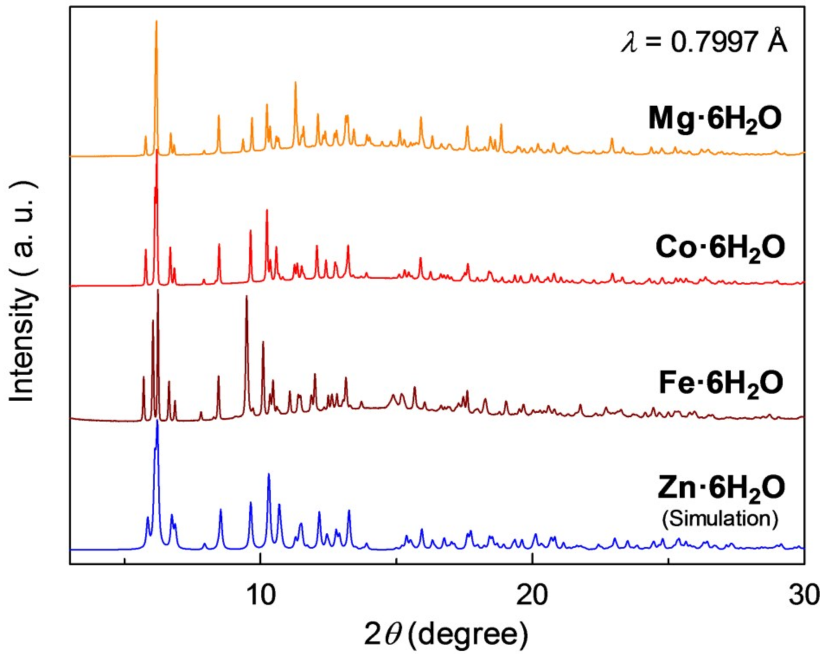

Figure S7 XRPD patterns of hexahydrate of **$M\cdot6H_2O$** (M = Fe, Co, Zn (simulation),[1] and Mg) at RT.

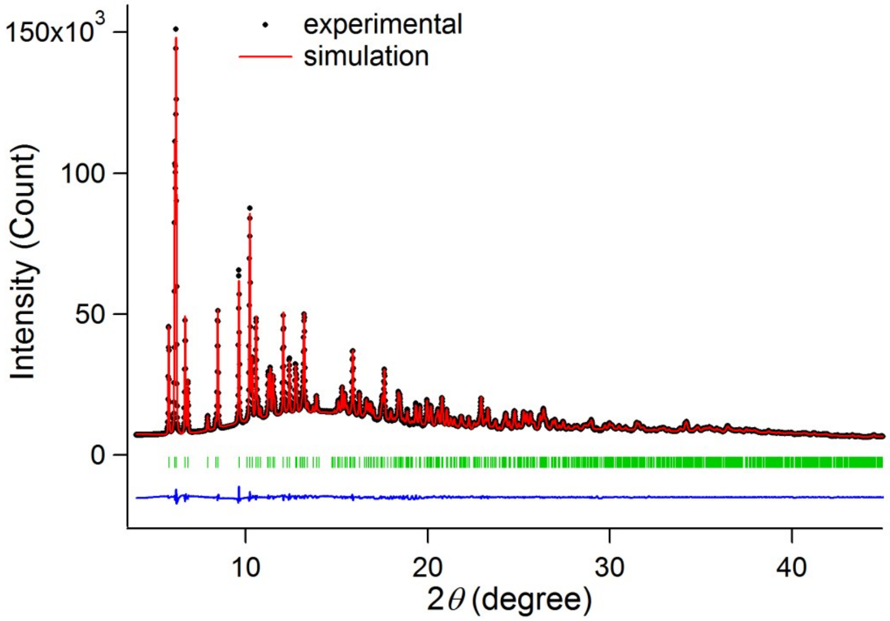

Figure S8 Results of Le Bail fittings for hexahydrate exemplified by **$Co\cdot6H_2O$**.

Table S6 Refined cell parameters of anhydrate of **$M\cdot6H_2O$** (M = Fe, Co, Zn,[1] and Mg) at RT.

	a / Å	b / Å	c / Å	α / °	β / °	γ / °	Space group	R_{wp}	Volume	Volume per formula
$Fe\cdot6H_2O$	8.3341(4)	16.0470(7)	9.3579(5)	90	113.554(2)	90	$P2_1/n$	4.75%	1147.2	573.6
$Co\cdot6H_2O$	8.3595(1)	15.8200(2)	9.3255(2)	90	114.675(1)	90	$P2_1/n$	1.91%	1120.66	560.33
$Zn\cdot6H_2O$	8.3549(3)	15.8063(5)	9.3155(3)	90	114.8555(5)	90	$P2_1/n$	2.06%	1116.25	558.13
$Mg\cdot6H_2O$	8.3589(2)	15.8385(4)	9.3247(3)	90	115.038(1)	90	$P2_1/n$	3.98%	1118.51	559.26

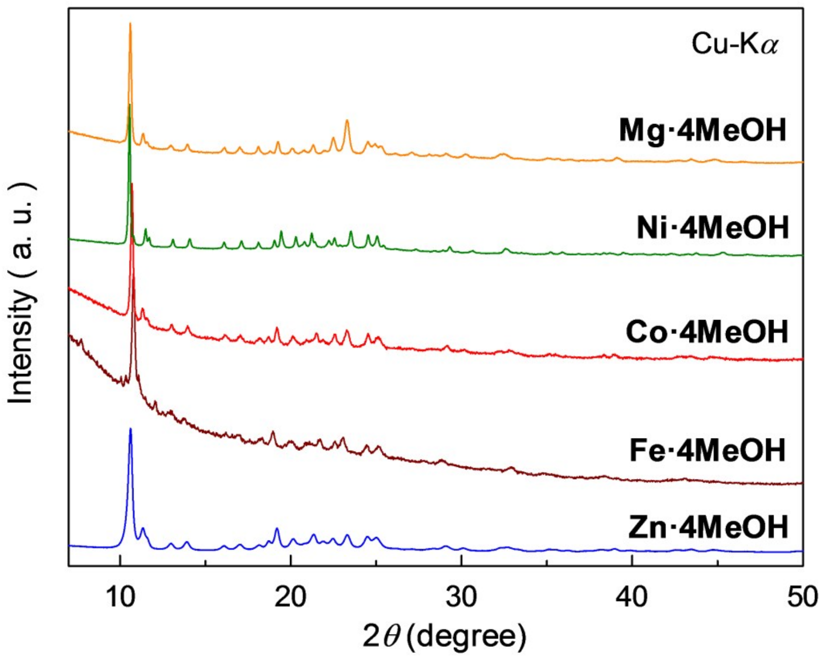

Figure S9 XRPD patterns of **M·4MeOH** (M = Fe, Co, Ni, Zn,[1] and Mg) at RT.

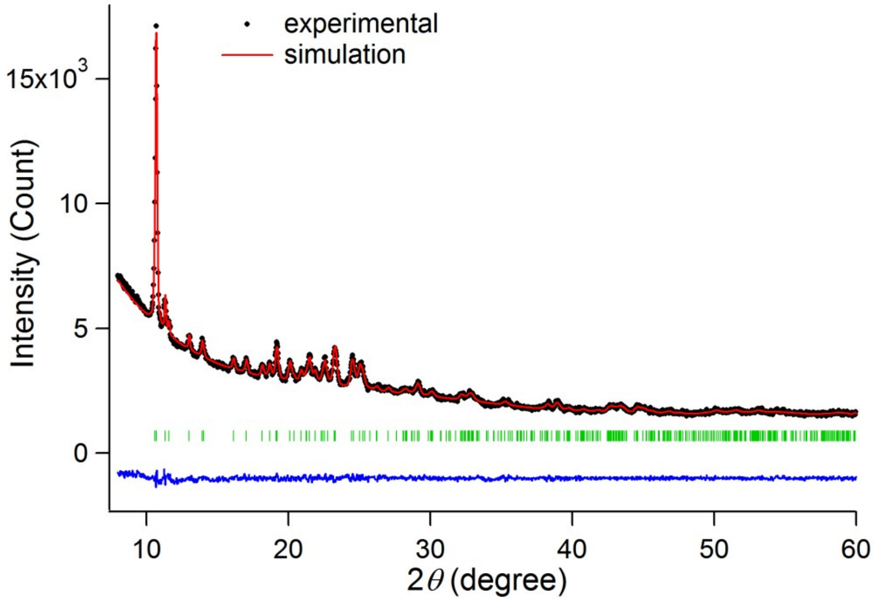

Figure S10 Results of Le Bail fittings for methanol-included samples exemplified by **Co·4MeOH**.

Table S7 Refined cell parameters of anhydrate of **M·4MeOH** (M = Fe, Co, Zn,[1] and Mg) at RT.

	a / Å	*b* / Å	*c* / Å	*α* / °	*β* / °	*γ* / °	Space group	R_{wp}	Volume	Volume per formula
Fe·4MeOH	8.845(3)	9.460(2)	9.057(3)	60.80(2)	72.70(3)	75.94(3)	*P*–1	2.60%	627.3	627.3
Co·4MeOH	8.722(1)	9.640(1)	9.091(1)	61.00(2)	73.22(2)	74.81(2)	*P*–1	2.29%	633.3	633.3
Ni·4MeOH	8.8260(5)	9.468(1)	8.9922(8)	60.723(8)	73.300(8)	75.519(8)	*P*–1	4.72%	622.4	622.4
Zn·4MeOH	8.7526(9)	9.5769(9)	9.0567(9)	60.998(9)	73.208(8)	75.051(9)	*P*–1	1.41%	629.31	629.31
Mg·4MeOH	8.8126(7)	9.599(1)	9.084(1)	60.90(1)	73.14(1)	74.93(1)	*P*–1	3.35%	636.1	636.1

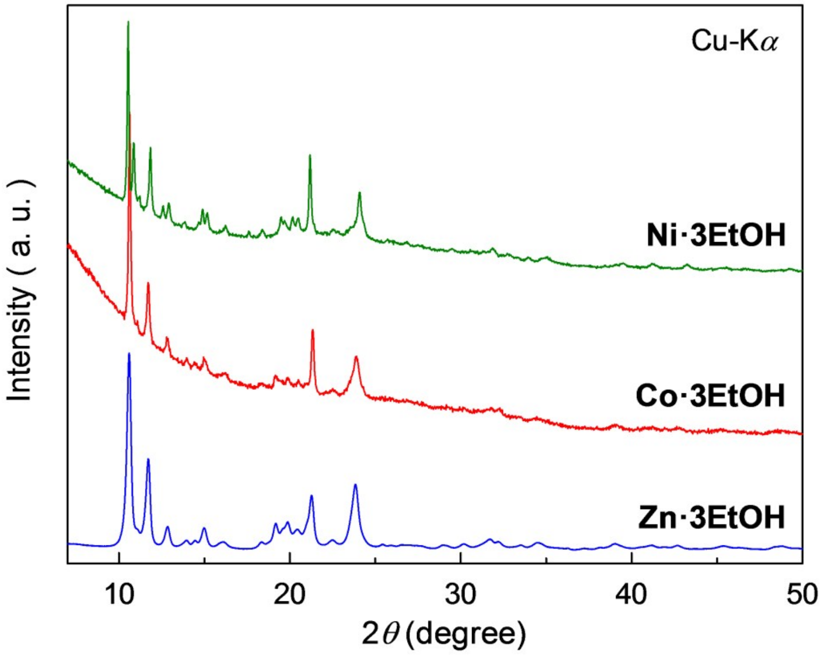

Figure S11 XRPD patterns of **M·3EtOH** (M = Co, Ni, and Zn[1]) at RT.

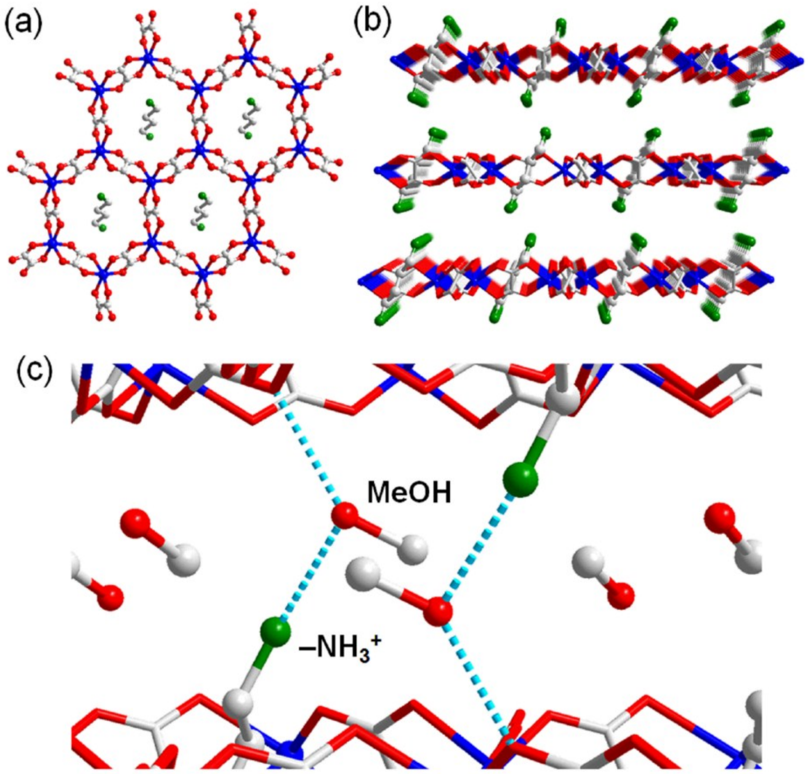

Figure S12 Crystal structure of **Zn·4MeOH**.[1] (a) Honeycomb layer framework and (b) a perspective view along a layer. The guest molecules have been omitted. (c) Hydrogen bonds around the guest molecules (Light blue dotted lines).

Reference

1. Sadakiyo, M.; Yamada, T.; Kitagawa, H. *J. Am. Chem. Soc.* **2011**, *133*, 11050–11053.
